# Protocol for analyzing protein ensemble structures from chemical cross-links using DynaXL

**DOI:** 10.1007/s41048-017-0044-9

**Published:** 2017-11-20

**Authors:** Zhou Gong, Zhu Liu, Xu Dong, Yue-He Ding, Meng-Qiu Dong, Chun Tang

**Affiliations:** 1 CAS Key Laboratory of Magnetic Resonance in Biological Systems, State Key Laboratory of Magnetic Resonance and Atomic Molecular Physics, and National Center for Magnetic Resonance at Wuhan, Wuhan Institute of Physics and Mathematics of the Chinese Academy of Sciences, Wuhan, 430071 China; 2 National Center for Magnetic Resonance at Wuhan, Wuhan Institute of Physics and Mathematics of the Chinese Academy of Sciences, Wuhan, 430071 China; 3 Department of Pharmacology, Institute of Neuroscience, Key Laboratory of Medical Neurobiology of the Ministry of Health of China, Zhejiang University School of Medicine, Hangzhou, 310057 China; 4 RNA Therapeutics Institute, University of Massachusetts Medical School, 368 Plantation Street, Worcester, MA 01605 USA; 5 National Institute of Biological Sciences, Beijing, 102206 China

**Keywords:** Chemical cross-linking, DynaXL, Ensemble refinement, Solvent accessible surface distance, Multi-domain protein, Protein–protein complex

## Abstract

Chemical cross-linking coupled with mass spectroscopy (CXMS) is a powerful technique for investigating protein structures. CXMS has been mostly used to characterize the predominant structure for a protein, whereas cross-links incompatible with a unique structure of a protein or a protein complex are often discarded. We have recently shown that the so-called over-length cross-links actually contain protein dynamics information. We have thus established a method called DynaXL, which allow us to extract the information from the over-length cross-links and to visualize protein ensemble structures. In this protocol, we present the detailed procedure for using DynaXL, which comprises five steps. They are identification of highly confident cross-links, delineation of protein domains/subunits, ensemble rigid-body refinement, and final validation/assessment. The DynaXL method is generally applicable for analyzing the ensemble structures of multi-domain proteins and protein–protein complexes, and is freely available at www.tanglab.org/resources.

**Electronic supplementary material:**

The online version of this article (10.1007/s41048-017-0044-9) contains supplementary material, which is available to authorized users.

## Introduction

**Fig. 1:**
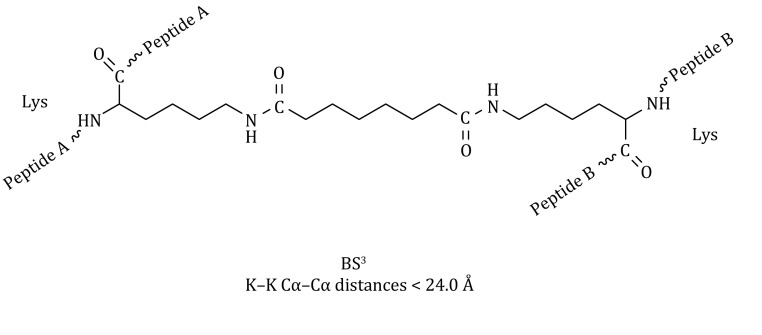
Chemical structure for the BS^3^ cross-link of two lysine residues in peptides A and B. The straight-line distance is less than 24 Å between the Cα atoms, and less than 24 Å between the Nζ atoms. Note that the lysine side-chain amine group switches from sp3 hybridization to sp2 hybridization

Chemical cross-linking coupled with mass spectroscopy (CXMS) has been used to characterize protein structures (Lasker *et al*. 2012; Walzthoeni *et al*. 2013; Politis *et al*. 2014). Different cross-linkers with various lengths and chemical properties are widely used in CXMS experiments. The commonly used cross-linking reagents include bis-sulfosuccinimidyl suberate (BS^3^), bis-sulfosuccinimidyl glutarate (BS^2^G), pimelic acid dihydrazide (PDH), and Leiker. (Leitner *et al*. 2014; Ding *et al*. 2016; Tan *et al*. 2016). Cross-linking reagents can react with specific amino acids in a protein, and two amino acids separated by a distance shorter than the length of the cross-linker can be theoretically cross-linked (Fig. 1). Mass spectrometry analysis is used to identify the cross-linked residues, which can be translated to inter-residue distance (Kahraman *et al*. 2013; Lossl *et al*. 2014). In addition, the protein does not have to be isotopically labeled, modified, or crystallized in CXMS experiments. The CXMS can also be used in conjunction with other methods, such as cryo-EM for characterizing the structures of protein machinery (Cheng *et al*. 2015a, b; Liu *et al*. 2015a, b).

Multi-domain proteins and protein–protein complexes often undergo conformational fluctuations (Liu *et al*. 2015a, b). As a result, the ensemble structures corresponding to multiple conformational states are required to fully depict protein dynamics. Protein structural characterization has been mostly focused on the predominant structure of a protein. Recently we and others have shown that the so-called over-length cross-links actually contain information about the alternative and often lowly populated conformational states of the protein (Shore *et al*. 2016). Based on the over-length cross-links, we have developed a computational approach called DynaXL to visualize protein dynamics. Using DynaXL, we were able to characterize the ensemble structures of protein–protein complexes, with the dissociation constant ranging from nanomolar to millimolar (Gong *et al*. 2015). We were also able to visualize open-to-closed movement of multi-domain proteins (Ding *et al*. 2017).

**Fig. 2:**
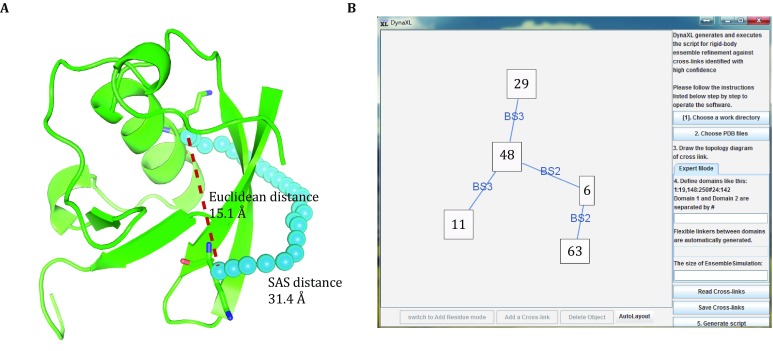
Key features of DynaXL program. **A** Comparison of Euclidean distance (denoted with yellow dashed line) and solvent accessible surface distance (denoted with cyan sphere) for Cβ atoms of the two Lys residues in the protein. **B** The graphical user interface for DynaXL, in which one residue can be cross-linked to multiple residues with different cross-linking reagents

An important feature of DynaXL is the use of solvent accessible surface distance (SASD) to describe the spatial relationship between cross-linked residues. As illustrated in Fig. 2A, the Euclidean straight-line distance between Cα atoms of Lys^29^ and Lys^6^ of Ubiquitin is 15.1 Å, while at 31.4 Å the SASD is much longer. As the cross-linker cannot penetrate through the protein and can only be at the protein surface, the SASD is a more realistic representation of the cross-linker and affords more stringent distance restraint. Explicit modeling of the cross-linker is incorporated into the software DynaXL with graphical interface (Fig. 2B). Here we will explain how to use DynaXL step by step.

## Overview of DynaXL Algorithm Design

**Fig. 3:**
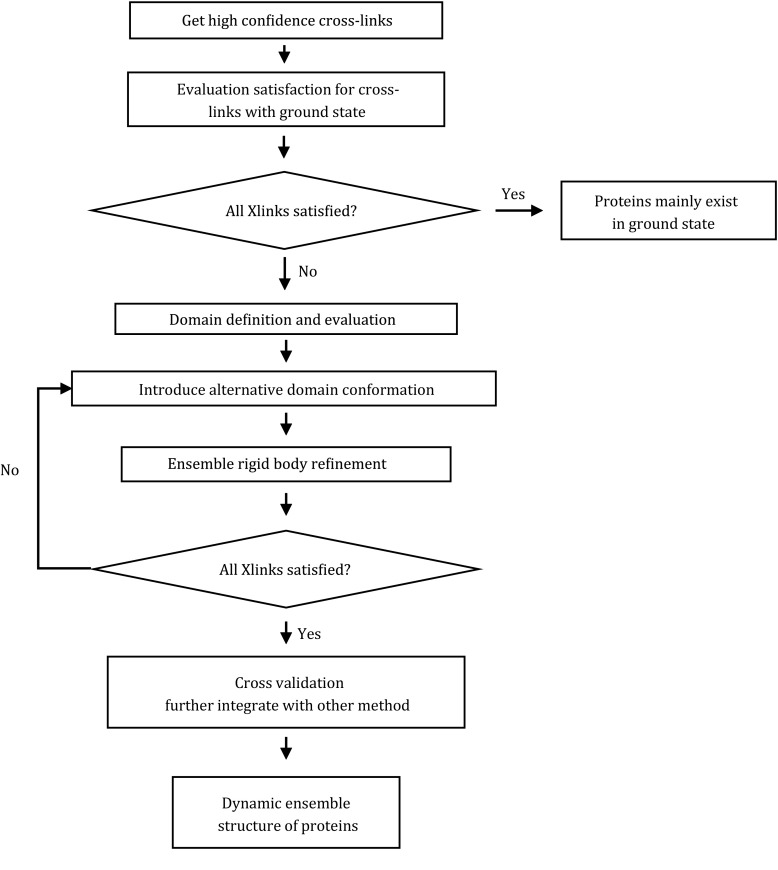
The overall flowchart for the refinement of protein ensemble structures using DynaXL

The DynaXL method contains four parts, as illustrated in Fig. 3.

1. Obtaining highly reliable cross-links using pLink (Yang *et al*. 2012). The acceptance criteria are the following: each spectrum should have an *E* value < 10^−3^, each cross-link must be identified by at least two spectra, and one of them should have an *E* value < 10^−8^.
2. Evaluating the conformational dynamics based on the CXMS data. The domains are defined based on the known structure of a protein and are treated as rigid bodies. For protein complexes, the monomers in the complex are treated as rigid bodies. The protein may solely exist in a single conformation if the known structure can already satisfy all experimental cross-links. Otherwise, there likely exist some alternative conformational states of the protein that give rise to the “over-length” cross-links.
3. Performing ensemble rigid-body refinement. One of the rigid bodies is kept fixed, and the other rigid body is subjected to translation and rotation. The number of structures in the ensemble is gradually increased if an *N* = 2 ensemble still cannot satisfy all cross-links. The optimal ensemble size is reached when all CXMS restraints are satisfied. There can be additional conformational state present for the protein system, which however is not captured and manifested by over-length cross-links.
4. Assessment of the ensemble structures, either by cross-validation or by corroboration from other experimental data.

## Materials and Equipment

### Software for cross-link identification

pLink (Yang *et al*. 2012) is the program used for querying a database containing the protein sequence and for identifying the cross-linked peptides.

### Software for protein structure modeling

Xplor-NIH (Schwieters *et al*. 2003), the software package for biomolecular structure refinement against experimental and knowledge-based restraints, is used here to identify an optimal ensemble structure that can account for all CXMS restraints.

AMBER 14 (Case *et al*. 2014),the molecular dynamic simulation package, is used here to refine the local conformation before ensemble refinement using Xplor-NIH.

Two programs, DynDom (Hayward *et al*. 1997; Hayward and Berendsen 1998) and ThreaDom (Xue *et al*. 2013; Wang *et al*. 2017), are used to define domain boundaries of multi-domain proteins.

PyMOL (the PyMOL Molecular Graphics System) is the software for illustrating and rendering protein structures.

## Summarized Procedure

1. To identify high-confidence cross-links from CXMS experiment;
2. To obtain the known structure from the PDB database;
3. To define domain boundaries;
4. To validate domain definition and evaluate local flexibility;
5. To classify and identify the cross-links (intra-domain vs. inter-domain, intramolecular vs. inter-molecular cross-links), and prepare the CXMS restraints table;
6. To prepare the starting structure files for Xplor-NIH;
7. To patch the cross-linker to the protein structure;
8. Ensemble rigid-body refinement against the CXMS restraints;
9. Cross-validation with a subset of cross-links;
10. To analyze and validate with other types of data.

## Procedure

Here, we use Ca^2+^-loaded calmodulin as an example to illustrate how DynaXL is used to account for all CXMS data and to afford the ensemble structures.

### Identification of cross-links

Intramolecular and inter-molecular cross-links can be differentiated by performing CXMS experiments on the sample containing equal molar amounts of unlabeled natural isotope abundance (light) and ^15^N-labeled proteins (heavy) (Ding *et al*. 2017). In this way, any cross-links arising from protein homodimer would contain both light and heavy peptides. Alternatively, the protein band corresponding to dimer (or monomer) can be excised from protein gel for mass spectrometry analysis. The intramolecular cross-links are further filtered with the following criteria using the software pLink.

1. False discovery rate cutoff of 0.05 is applied and followed by an *E* value cutoff rate (Yang *et al*. 2012) of 10^−3^ at the spectrum level;
2. Spectral count ≥ 2 and the best *E* value < 10^−8^ for each pair of cross-link.

The cross-link spectra that pass the false discovery rate (FDR) cutoff are further filtered with these requirements: (A) each spectrum should have an *E* value of < 10^−3^, and (B) each cross-link should be identified in at least two spectra.

### Assessment of the predominant structure of the protein

The structure of the predominant conformational state of the protein under investigation can be downloaded from the PDB. For proteins without known structure, the structure can be modeled from homology modeling (Marti-Renom *et al*. 2000), domain threading (Yang *et al*. 2015), or fragment splicing (Rohl *et al*. 2004).

The definition of protein domain boundary is performed with protein domain motion analysis (Hayward *et al*. 1997; Hayward and Berendsen 1998), multi-threading alignment (Xue *et al*. 2013; Belsom *et al*. 2016), or the assessment of evolutionary relationships (Cheng *et al*. 2014, 2015a, b). It should be noted that the definition of protein domain is not immutable, but will be amended based on further calculation and analysis (see below). For protein complexes, each subunit in the complex is treated as an individual rigid body.

### Evaluation of protein local flexibility

1. Structure completion. The atomic information is often missing in the PDB file, which is especially true for the X-ray structure. The missing parts include flexible loops and N- and C-terminal tails. In addition, hydrogen atoms are usually absent in relatively low-resolution structures. To complete the missing residues, *e.g.*, the first three amino acids in calmodulin PDB structure 1CLL, the build-residue function in PyMOL software is used. To complete the missing atoms, *e.g.*, side-chain atoms or hydrogen atoms, either PyMOL build-residue module or MD simulation software AMBER can be used.
2. Flexibility evaluation. MD simulation using software AMBER can provide local flexibility for different parts of the protein upon assessing the fluctuation over time. The flexibility can also be assessed from crystal *B*-factors (Shore *et al*. 2016) and from NMR heteronuclear NOE values (Shore *et al*. 2016).

### Identification and classification of the cross-links

Based on the known structure and domain definitions, the intramolecular cross-links can be classified into two categories including intra-domain and inter-domain ones. The intra-domain cross-links can also be used to confirm the definition of domains. For a protein complex, the cross-links are categorized as intramolecular and inter-molecular cross-links. If the known structure cannot satisfy all intramolecular inter-domain cross-links, there can be two possibilities:

1. The discrepancy between the theoretical solvent-accessible inter-residue surface distance and the maximum length of the cross-linker is small, and the over-length cross-links can be attributed to local dynamic of the protein.
2. The discrepancy between the theoretical solvent-accessible inter-residue surface distance and the maximum length of the cross-linker is large. Local dynamics alone cannot account for all the over-length cross-links. Therefore, the protein has to undergo collective domain movement, and further computational analysis is warranted.

### Ensemble rigid-body refinement against CXMS restraints

The ensemble rigid-body refinement against CXMS restraints is performed when the predominant conformation or the known structure of the protein cannot satisfy all intramolecular inter-domain cross-links identified with high confidence. The protein domains are treated as rigid bodies, and their relative orientations are optimized on the basis of explicitly represented CXMS distance restraints. Similar approach is used to optimize the ensemble structures of protein–protein complexes. The details of the process are as follows.

#### Prepare the initial structure for Xplor-NIH

The structure refinement process against the CXMS restraints is conducted with the use of Xplor-NIH software. The Xplor-NIH requires a PDB file (providing atomic coordinate information) and a PSF file (providing structural connection information and other parameters) as the initial input. The user should pay attention to the following when preparing the input files:

(A) Different programs may have different atomic naming rules (especially for hydrogen atoms). Therefore, one should first remove all the hydrogen atoms, and use the Xplor-NIH script to re-protonate the protein.
(B) For assessment of protein local dynamics and domain boundaries, it may be necessary to re-number the residues of the protein. The first residue handled by AMBER software is always 1. Please refer to the respective manual for the software used.
(C) The Xplor-NIH will add an extra oxygen atom for the last residue and rename the last two oxygen atoms as OT1 and OT2 in the PSF file. As a result, the PSF file may be inconsistent with the PDB file provided. A quick solution is to duplicate the last line in the PDB file and to name the last two atoms as OT1 and OT2 as follows:

**Table:** 

ATOM	2261	C	LYS	148	12.612	14.659	− 4.066	1.00	0.00
ATOM	2262	OT1	LYS	148	12.190	15.449	− 4.923	1.00	0.00
ATOM	2263	OT2	LYS	148	12.190	15.449	− 4.923	1.00	0.00

#### Patch the cross-linker to protein

In DynaXL, the cross-linker is explicitly modeled onto the protein structure. For a pair of cross-linked lysine residues, the cross-linker is patched to one of the residues with the formation of an isopeptide bond. Similar approach can also be used to patch other cross-linking reagent with different reactivity to different types of protein residues. The patching process is done in these steps:

(A) Presented in this Protocol, we use two common cross-linkers BS^2^G and BS^3^, whose PDB and PSF files are provided in “Supplementary material.” For other types of cross-linking reagents, the PDB files can be generated using the build function in PyMOL, and the corresponding parameter file can be obtained from online servers like HIC-Up (Kleywegt 2007) or PRODRG (Schuttelkopf and van Aalten 2004).
(B) For subsequent structure optimization, the cross-linker is only patched to one of the two cross-linked residues or to one of the protein domains. We have found that patching the cross-linker to either domain affords essentially the same results. The peptide bonds are formed between the side-chain of the protein and the cross-linkers, thus requiring the modification of the corresponding atoms. For example, as the nitrogen atom (N_ζ_) of Lys side-chain is connected with three hydrogen atoms, it is necessary to remove the two extra hydrogen atoms, and the atom types for the remaining nitrogen ant hydrogen atoms are modified accordingly.
(C) The segment ID is another important distinguisher in Xplor-NIH in addition to the residue ID, *i.e.,* residues with the same residue ID values (residue number) and with different segment ID values correspond to different residues. It happens when a residue can be cross-linked to different residues in the opposite domain. Thus, we assign the cross-links at the same residue with different segment ID values. Physically, the multiple cross-links involving same residues should take place one at a time, and accordingly the *iso*-residue cross-links can overlap with each other without incurring van der Waals clashes during the refinement.

#### Preparation of the CXMS restraints table

With the cross-linker patched to one domain (or one subunit), the cross-linking process is simulated by enforcing a distance restraint between the end of the cross-linker and the reactive group of the other cross-linked residue. Specifically, it is achieved by constraining the distance between the carbonyl atom in the cross-linker and Lys N_ζ_ atom ranging from 1.3 Å (covalent bond length) to 5 Å (the sum of the VDW radius of both the carbon atom and the nitrogen).

#### Simulated annealing refinement

When all the input files and constraint files are prepared, a user can start the ensemble rigid-body refinement against the CXMS restraints. As mentioned above, when the given structure cannot satisfy all experimental high-confidence cross-links, the ensemble refinement based on CXMS restraints can be performed. The over-length cross-links capture one or more alternative conformational states. The ensemble refinement process starts with an *N* = 2 ensemble that comprises the predominant conformation and the alternative one. An additional conformer is included if the *N* = 2 ensemble cannot satisfy all the cross-links. The process is repeated until the experimental data are fully accounted for.

Here, we treat the different domains of the protein as rigid bodies, and the local conformational changes within each domain are not considered. The connecting loop residues between the domains are given full torsion angle freedom. As the domain movement is relative, one domain is kept fixed, and the other domain(s) are grouped together and are allowed to freely rotate and to translate with respect to the fixed domain. The fixing/grouping is implemented using the following script in Xplor-NIH.

- dyn.fix (“““ segid “ “ and resi 1:77 “““)
- dyn.group (“““ segid ALT0 and resi 82:148 “““)
- dyn.group (“““ segid ALT1 and resi 82:148 “““)

Here, the N-terminal domain including residues 1–77 of the calmodulin is fixed, and the C-terminal domain including residues 82–148 moves as a rigid body. In addition, the flexible loop region between the including residues 78–81 has full torsional freedom. In the Xplor-NIH script shown above, note that there are two different conformers for the C-terminal domain, marked with segment ID ALT0 and ALT1. The two conformers correspond to the two conformational states of calmodulin. To speed up the computation, the non-bonded van der Waals interactions within each rigid body are not considered and calculated. This is implemented using the following statement.

Constraints:

- inter = (segid “ “) (segid ALT0 or resi 82:148)
- inter = (segid “ “) (segid ALT1 or resi 82:148)
- inter = (segid ALT0 and resi 78:82) (segid “ “ and segid ALT0)
- inter = (segid ALT1 and resi 78:82) (segid “ “ and segid ALT1)
- weights * 1 end end

In the ensemble refinement, ambiguous distance restraints are employed, and the back-calculated distance *R* is defined as

$$ R = \left( {\mathop \sum \limits_{k = 1}^{N} r_{k}^{ - 6} } \right)^{ - 1/6}. $$

In which *r*_k_ is the distance between the two atoms (nitrogen atom from the lysine residue and carbonyl atom from the cross-linker as discussed before) in conformer *k*, and *N* is the number of conformers in the ensemble. An exponential factor of − 6 is used here, and as a result the < *r* ^−6^ > averaged distance is heavily biased towards the shortest distance.

The ensemble refinement is carried out by simulated annealing. The system is heated to a relatively high temperature and then slowly cooled down. The structure is refined against the CXMS restraints during the cooling process. The computational process is repeated many times for effective sampling. Finally, the structures with no CXMS violation (satisfying all cross-links) and low energy (no atomic overlap) are selected for further analysis.

We have found that the explicit representation of cross-linker not only provides more realistic and stringent restraints, but also allows better convergence for the ensemble structures, as compared to straight-line Euclidean distance restraints.

### Cross-validation with a subset of cross-links

The cross-validation process is performed to verify the accuracy of the ensemble structures. In detail, a subset of CXMS restraints is removed, and the remaining cross-links are used for the ensemble refinement as described above. The ensemble structures generated with a subset of the restraints are evaluated and the CXMS restraints excluded in the refinement should be cross-validated.

### Analysis and validation with other types of experimental data

The over-length cross-links capture protein alternative conformations in solution. The ensemble structures obtained by refining against the CXMS restraints may be compared to those obtained from other biochemical and biophysical methods, such as paramagnetic relaxation enhancement (Tang *et al*. 2006) and small-angle X-ray scattering (Schneidman-Duhovny *et al*. 2012; Kikhney and Svergun 2015).

## Limitations of the DynaXL Method

The ensemble structures obtained based on the CXMS restraints may suffer from certain limitations as described below.

### False identifications

Due to the quality of the mass spectra, false identification of the cross-links may occur. In other words, the experiment may identify incorrect cross-links, even though stringent criteria are applied when selecting high-confidence cross-links. Multiple technical and biological repeats are necessary to minimize false identifications.

### Insufficient number of restraints

There may not be a large number cross-links identified with high confidence that can be used as the restraints. Certainly more restraints would enable a researcher to better refine the structure and to discover discrepancy within the restraints. However, it has been shown that the structural model of a protein complex can be obtained from just a single inter-molecular cross-linking restraint (Gong *et al*. 2015). Thus, the DynaXL approach may only identify the minimum number of ensemble structures that can account for all available CXMS restraints. Should there are more conformational states that elude cross-linking reactions, DynaXL cannot uncover.

### Over-fitting problem

The ensemble size may have to be increased to account for all the cross-links. Additional conformers introduce additional parameters, which may lead to over-fitting. It is also possible that some over-length cross-links can be satisfied by intra-domain dynamics without the invocation of domain movement. Thus, cross-validation is important.

## Future Perspective

CXMS has been increasingly used for protein structure modeling. Here, we present the detailed protocol using DynaXL for explicitly modeling the cross-links and characterization of protein ensemble structures. The chemical cross-linking as well photo-cross-linking are rapidly evolving (Chiang *et al*. 2016), and new types of cross-linking reagents (Brodie *et al*. 2016) with various linker lengths and reactivity are becoming increasingly available, which can afford more spatial information between protein residues. In the age of integrative structural biology, protein ensemble structures can be better visualized with the joint refinement against multiple types of experimental inputs including but not limited to NMR, cryo-EM, and FRET.

## Electronic Supplementary Material

Below is the link to the electronic supplementary material.

Supplementary material 1 (PDF 45 kb)
