# Supplementary material for: Protocol for analyzing protein ensemble structures from chemical cross-links using DynaXL
Source: Biophys Rep. 2017 Nov 20;3(4):100–8. doi: 10.1007/s41048-017-0044-9 (PMC5719800; doi:10.1007/s41048-017-0044-9)
Supplement: Supplementary file 1 — Supplementary material 1 (PDF 45 kb) [file 41048_2017_44_MOESM1_ESM.pdf]

Supplementary material

- PDB coordinate for BS^2G

ATOM	1	C01	BS2	503	−9.171	0.642	0.321	0.00	0.00	C
ATOM	2	C05	BS2	503	−6.096	−1.442	−3.070	0.00	0.00	C
ATOM	3	O11	BS2	503	−9.984	0.176	−0.429	0.00	0.00	O1−
ATOM	4	O51	BS2	503	−5.267	−2.310	−3.059	0.00	0.00	O1−
ATOM	5	C02	BS2	503	−7.701	0.470	−0.105	0.00	0.00	C
ATOM	6	C03	BS2	503	−7.633	−0.401	−1.373	0.00	0.00	C
ATOM	7	O12	BS2	503	−9.521	1.208	1.348	0.00	0.00	O
ATOM	8	O52	BS2	503	−6.822	−1.285	−4.042	0.00	0.00	O
ATOM	9	C04	BS2	503	−6.163	−0.575	−1.799	0.00	0.00	C
ATOM	10	H21	BS2	503	−7.143	−0.013	0.698	0.00	0.00	H
ATOM	11	H22	BS2	503	−7.266	1.448	−0.312	0.00	0.00	H
ATOM	12	H32	BS2	503	−8.070	−1.378	−1.167	0.00	0.00	H
ATOM	13	H42	BS2	503	−5.725	0.402	−2.003	0.00	0.00	H
ATOM	14	H31	BS2	503	−8.188	0.083	−2.176	0.00	0.00	H
ATOM	15	H41	BS2	503	−5.608	−1.061	−0.996	0.00	0.00	H
END										

- Parameters for BS^2G

```
Remarks You *MUST* check/edit MASSes and CHARges !!!
Remarks Check DONOrs and ACCEptors
Remarks Verify IMPRopers yourself
Remarks DIHEdrals which are not flat are commented out

 set echo=false end

 { Note: edit masses if necessary }
{
 MASS CX1      12.01100 ! assuming C -> 12.01100 + 1.008 * 0 (Hs)
 MASS CX2      12.01100 ! assuming C -> 12.01100 + 1.008 * 0 (Hs)
 MASS OX3      15.99900 ! assuming O -> 15.99900 + 1.008 * 0 (Hs)
 MASS OX4      15.99900 ! assuming O -> 15.99900 + 1.008 * 0 (Hs)
 MASS CX5      12.01100 ! assuming C -> 12.01100 + 1.008 * 0 (Hs)
 MASS CX6      12.01100 ! assuming C -> 12.01100 + 1.008 * 0 (Hs)
 MASS OX7      15.99900 ! assuming O -> 15.99900 + 1.008 * 0 (Hs)
 MASS OX8      15.99900 ! assuming O -> 15.99900 + 1.008 * 0 (Hs)
 MASS CX9      12.01100 ! assuming C -> 12.01100 + 1.008 * 0 (Hs)
 MASS HX10     1.008
```

```
  MASS HX11      1.008
  MASS HX12      1.008
  MASS HX13      1.008
  MASS HX14      1.008
  MASS HX15      1.008
}

  autogenerate angles=true end

RESIdue BS2

  { Note: electrostatics should normally not be used in }
  { crystallographic refinement since it can produce }
  { artefacts. For this reason, all charges are set to }
  { zero by default. Edit them if necessary }

GROUp
  ATOM    C01    TYPE C       CHARge    0.0    END ! Nr of Hs =    0
  ATOM    C05    TYPE C       CHARge    0.0    END ! Nr of Hs =    0
  ATOM    O11    TYPE OC      CHARge    0.0    END ! Nr of Hs =    0
  ATOM    O51    TYPE OC      CHARge    0.0    END ! Nr of Hs =    0
  ATOM    C02    TYPE CT      CHARge    0.0    END ! Nr of Hs =    0
  ATOM    C03    TYPE CT      CHARge    0.0    END ! Nr of Hs =    0
  ATOM    O12    TYPE OC      CHARge    0.0    END ! Nr of Hs =    0
  ATOM    O52    TYPE OC      CHARge    0.0    END ! Nr of Hs =    0
  ATOM    C04    TYPE CT      CHARge    0.0    END ! Nr of Hs =    0
  ATOM    H21    TYPE HA      CHARge    0.0    END ! Nr of Hs =    0
  ATOM    H22    TYPE HA      CHARge    0.0    END ! Nr of Hs =    0
  ATOM    H32    TYPE HA      CHARge    0.0    END ! Nr of Hs =    0
  ATOM    H42    TYPE HA      CHARge    0.0    END ! Nr of Hs =    0
  ATOM    H31    TYPE HA      CHARge    0.0    END ! Nr of Hs =    0
  ATOM    H41    TYPE HA      CHARge    0.0    END ! Nr of Hs =    0

  BOND    C01    O11      BOND    C01    C02     BOND    C01    O12      BOND    C05    O51
  BOND    C05    O52      BOND    C05    C04     BOND    C02    C03      BOND    C02    H21
  BOND    C02    H22      BOND    C03    C04     BOND    C03    H32      BOND    C03    H31
  BOND    C04    H42      BOND    C04    H41

  { Note: edit these DIHEdrals if necessary }

    DIHEdral    O11    C01    C02    C03 ! angle 1
    DIHEdral    C01    C02    C03    C04 ! angle 2
    DIHEdral    C02    C03    C04    C05 ! angle 3
    DIHEdral    C03    C04    C05    O51 ! angle 4
```

```
{
! DIHEdral   O11   C01   C02   H21 ! flexible dihedral ???   −125.24
! DIHEdral   O11   C01   C02   H22 ! flexible dihedral ???    114.77
  DIHEdral   O12   C01   C02   C03 ! flat ? (180 degrees = trans)    174.68
! DIHEdral   O12   C01   C02   H21 ! flexible dihedral ???     54.67
! DIHEdral   O12   C01   C02   H22 ! flexible dihedral ???    −65.33
  DIHEdral   C01   C02   C03   C04 ! flat ? (180 degrees = trans)    180.06
! DIHEdral   C01   C02   C03   H32 ! flexible dihedral ???    −59.88
! DIHEdral   C01   C02   C03   H31 ! flexible dihedral ???     60.12
! DIHEdral   H21   C02   C03   C04 ! flexible dihedral ???    −59.89
! DIHEdral   H21   C02   C03   H32 ! flexible dihedral ???     60.16
  DIHEdral   H21   C02   C03   H31 ! flat ? (180 degrees = trans)    180.17
! DIHEdral   H22   C02   C03   C04 ! flexible dihedral ???     60.08
  DIHEdral   H22   C02   C03   H32 ! flat ? (180 degrees = trans)    180.13
! DIHEdral   H22   C02   C03   H31 ! flexible dihedral ???    −59.86
  DIHEdral   C02   C03   C04   C05 ! flat ? (180 degrees = trans)    180.20
! DIHEdral   C02   C03   C04   H42 ! flexible dihedral ???    −59.82
! DIHEdral   C02   C03   C04   H41 ! flexible dihedral ???     60.13
! DIHEdral   H32   C03   C04   C05 ! flexible dihedral ???     60.14
  DIHEdral   H32   C03   C04   H42 ! flat ? (180 degrees = trans)    180.12
! DIHEdral   H32   C03   C04   H41 ! flexible dihedral ???    −59.93
! DIHEdral   H31   C03   C04   C05 ! flexible dihedral ???    −59.83
! DIHEdral   H31   C03   C04   H42 ! flexible dihedral ???     60.15
  DIHEdral   H31   C03   C04   H41 ! flat ? (180 degrees = trans)    180.10
}

  { Note: edit these IMPRopers if necessary }
  IMPRoper   C01   C02   O11   O12 ! chirality or flatness improper      0.05
  IMPRoper   C05   C04   O51   O52 ! flatness improper     −0.01

{
  IMPRoper   C02   C01   C03   H21 ! chirality or flatness improper     30.36
  IMPRoper   C03   C02   C04   H32 ! chirality or flatness improper    −30.35
  IMPRoper   C04   C05   C03   H42 ! chirality or flatness improper     30.37
}
{
! DIHEdral   O11   C01   C02   H21 ! flexible dihedral ???   −125.24
! DIHEdral   O11   C01   C02   H22 ! flexible dihedral ???    114.77
  DIHEdral   O12   C01   C02   C03 ! flat ? (180 degrees = trans)    174.68
! DIHEdral   O12   C01   C02   H21 ! flexible dihedral ???     54.67
! DIHEdral   O12   C01   C02   H22 ! flexible dihedral ???    −65.33
  DIHEdral   C01   C02   C03   C04 ! flat ? (180 degrees = trans)    180.06
! DIHEdral   C01   C02   C03   H32 ! flexible dihedral ???    −59.88
! DIHEdral   C01   C02   C03   H31 ! flexible dihedral ???     60.12
```

```
! DIHEdral    H21    C02    C03    C04 ! flexible dihedral ???      −59.89
! DIHEdral    H21    C02    C03    H32 ! flexible dihedral ???        60.16
  DIHEdral    H21    C02    C03    H31 ! flat ? (180 degrees = trans)      180.17
! DIHEdral    H22    C02    C03    C04 ! flexible dihedral ???        60.08
  DIHEdral    H22    C02    C03    H32 ! flat ? (180 degrees = trans)      180.13
! DIHEdral    H22    C02    C03    H31 ! flexible dihedral ???      −59.86
  DIHEdral    C02    C03    C04    C05 ! flat ? (180 degrees = trans)      180.20
! DIHEdral    C02    C03    C04    H42 ! flexible dihedral ???      −59.82
! DIHEdral    C02    C03    C04    H41 ! flexible dihedral ???        60.13
! DIHEdral    H32    C03    C04    C05 ! flexible dihedral ???        60.14
  DIHEdral    H32    C03    C04    H42 ! flat ? (180 degrees = trans)      180.12
! DIHEdral    H32    C03    C04    H41 ! flexible dihedral ???      −59.93
! DIHEdral    H31    C03    C04    C05 ! flexible dihedral ???      −59.83
! DIHEdral    H31    C03    C04    H42 ! flexible dihedral ???        60.15
  DIHEdral    H31    C03    C04    H41 ! flat ? (180 degrees = trans)      180.10
}

  { Note: edit these IMPRopers if necessary }
  IMPRoper    C01    C02    O11    O12 ! chirality or flatness improper          0.05
  IMPRoper    C05    C04    O51    O52 ! flatness improper        −0.01

{
  IMPRoper    C02    C01    C03    H21 ! chirality or flatness improper        30.36
  IMPRoper    C03    C02    C04    H32 ! chirality or flatness improper      −30.35
  IMPRoper    C04    C05    C03    H42 ! chirality or flatness improper        30.37
}
{ Note: edit any DONOrs and ACCEptors if necessary }
{
  ACCEptor    O11    C01
  ACCEptor    O51    C05
  ACCEptor    O12    C01
  ACCEptor    O52    C05
}
END { RESIdue BS2 }
```

- PDB coordinate for BS[3]

```
ATOM      1   C08 BS3    601      −2.977   −2.604   −5.099   0.00   0.00      C
ATOM      2   C01 BS3    601      −9.130    0.649    0.362   0.00   0.00      C
ATOM      3   O81 BS3    601      −2.171   −2.488   −4.217   0.00   0.00      O1−
ATOM      4   O11 BS3    601      −9.936    0.214   −0.414   0.00   0.00      O1−
ATOM      5   C02 BS3    601      −7.657    0.503   −0.062   0.00   0.00      C
ATOM      6   C03 BS3    601      −7.569   −0.392   −1.312   0.00   0.00      C
ATOM      7   O82 BS3    601      −2.617   −2.823   −6.248   0.00   0.00      O
```

ATOM	8	O12	BS3	601	−9.490	1.168	1.410	0.00	0.00	O
ATOM	9	C04	BS3	601	−6.096	−0.541	−1.735	0.00	0.00	C
ATOM	10	C05	BS3	601	−6.010	−1.428	−2.992	0.00	0.00	C
ATOM	11	C06	BS3	601	−4.536	−1.580	−3.413	0.00	0.00	C
ATOM	12	C07	BS3	601	−4.450	−2.459	−4.674	0.00	0.00	C
ATOM	13	H52	BS3	601	−6.429	−2.410	−2.774	0.00	0.00	H
ATOM	14	H21	BS3	601	−7.088	0.050	0.751	0.00	0.00	H
ATOM	15	H22	BS3	601	−7.244	1.486	−0.290	0.00	0.00	H
ATOM	16	H31	BS3	601	−8.137	0.061	−2.124	0.00	0.00	H
ATOM	17	H32	BS3	601	−7.982	−1.375	−1.084	0.00	0.00	H
ATOM	18	H41	BS3	601	−5.529	−1.001	−0.926	0.00	0.00	H
ATOM	19	H42	BS3	601	−5.681	0.442	−1.956	0.00	0.00	H
ATOM	20	H62	BS3	601	−4.115	−0.597	−3.625	0.00	0.00	H
ATOM	21	H51	BS3	601	−6.573	−0.964	−3.802	0.00	0.00	H
ATOM	22	H61	BS3	601	−3.974	−2.048	−2.605	0.00	0.00	H
ATOM	23	H71	BS3	601	−5.017	−1.994	−5.481	0.00	0.00	H
ATOM	24	H72	BS3	601	−4.866	−3.443	−4.459	0.00	0.00	H

END

- Parameters for BS^3

```
Remarks You *MUST* check/edit MASSes and CHARges !!!
Remarks Check DONOrs and ACCEptors
Remarks Verify IMPRopers yourself
Remarks DIHEdrals which are not flat are commented out

 set echo=false end

 { Note: edit masses if necessary }
{
 MASS CX1      12.01100 ! assuming C -> 12.01100 + 1.008 * 0 (Hs)
 MASS CX2      12.01100 ! assuming C -> 12.01100 + 1.008 * 0 (Hs)
 MASS OX3      15.99900 ! assuming O -> 15.99900 + 1.008 * 0 (Hs)
 MASS OX4      15.99900 ! assuming O -> 15.99900 + 1.008 * 0 (Hs)
 MASS CX5      12.01100 ! assuming C -> 12.01100 + 1.008 * 0 (Hs)
 MASS CX6      12.01100 ! assuming C -> 12.01100 + 1.008 * 0 (Hs)
 MASS OX7      15.99900 ! assuming O -> 15.99900 + 1.008 * 0 (Hs)
 MASS OX8      15.99900 ! assuming O -> 15.99900 + 1.008 * 0 (Hs)
 MASS CX9      12.01100 ! assuming C -> 12.01100 + 1.008 * 0 (Hs)
 MASS CX10     12.01100 ! assuming C -> 12.01100 + 1.008 * 0 (Hs)
 MASS CX11     12.01100 ! assuming C -> 12.01100 + 1.008 * 0 (Hs)
 MASS CX12     12.01100 ! assuming C -> 12.01100 + 1.008 * 0 (Hs)
 MASS FX13     18.99800 ! assuming F -> 18.99800 + 1.008 * 0 (Hs)
```

```
  MASS FX14      18.99800 ! assuming F -> 18.99800 + 1.008 * 0 (Hs)
  MASS FX15      18.99800 ! assuming F -> 18.99800 + 1.008 * 0 (Hs)
  MASS FX16      18.99800 ! assuming F -> 18.99800 + 1.008 * 0 (Hs)
  MASS FX17      18.99800 ! assuming F -> 18.99800 + 1.008 * 0 (Hs)
  MASS FX18      18.99800 ! assuming F -> 18.99800 + 1.008 * 0 (Hs)
  MASS FX19      18.99800 ! assuming F -> 18.99800 + 1.008 * 0 (Hs)
  MASS FX20      18.99800 ! assuming F -> 18.99800 + 1.008 * 0 (Hs)
  MASS FX21      18.99800 ! assuming F -> 18.99800 + 1.008 * 0 (Hs)
  MASS FX22      18.99800 ! assuming F -> 18.99800 + 1.008 * 0 (Hs)
  MASS FX23      18.99800 ! assuming F -> 18.99800 + 1.008 * 0 (Hs)
  MASS FX24      18.99800 ! assuming F -> 18.99800 + 1.008 * 0 (Hs)
}
  autogenerate angles=true end
RESIdue BS3

  { Note: electrostatics should normally not be used in }
  { crystallographic refinement since it can produce }
  { artefacts. For this reason, all charges are set to }
  { zero by default. Edit them if necessary }
GROUp

  ATOM    C08    TYPE C      CHARge    0.0    END ! Nr of Hs =    0
  ATOM    C01    TYPE C      CHARge    0.0    END ! Nr of Hs =    0
  ATOM    O81    TYPE OC     CHARge    0.0    END ! Nr of Hs =    0
  ATOM    O11    TYPE OC     CHARge    0.0    END ! Nr of Hs =    0
  ATOM    C02    TYPE CT     CHARge    0.0    END ! Nr of Hs =    0
  ATOM    C03    TYPE CT     CHARge    0.0    END ! Nr of Hs =    0
  ATOM    O82    TYPE OC     CHARge    0.0    END ! Nr of Hs =    0
  ATOM    O12    TYPE OC     CHARge    0.0    END ! Nr of Hs =    0
  ATOM    C04    TYPE CT     CHARge    0.0    END ! Nr of Hs =    0
  ATOM    C05    TYPE CT     CHARge    0.0    END ! Nr of Hs =    0
  ATOM    C06    TYPE CT     CHARge    0.0    END ! Nr of Hs =    0
  ATOM    C07    TYPE CT     CHARge    0.0    END ! Nr of Hs =    0

  ATOM    H52    TYPE HA    CHARge    0.0    END ! Nr of Hs =    0
  ATOM    H21    TYPE HA    CHARge    0.0    END ! Nr of Hs =    0
  ATOM    H22    TYPE HA    CHARge    0.0    END ! Nr of Hs =    0
  ATOM    H31    TYPE HA    CHARge    0.0    END ! Nr of Hs =    0
  ATOM    H32    TYPE HA    CHARge    0.0    END ! Nr of Hs =    0
  ATOM    H41    TYPE HA    CHARge    0.0    END ! Nr of Hs =    0
  ATOM    H42    TYPE HA    CHARge    0.0    END ! Nr of Hs =    0
  ATOM    H62    TYPE HA    CHARge    0.0    END ! Nr of Hs =    0
  ATOM    H51    TYPE HA    CHARge    0.0    END ! Nr of Hs =    0
  ATOM    H61    TYPE HA    CHARge    0.0    END ! Nr of Hs =    0
```

```
  ATOM    H71    TYPE HA    CHARge    0.0    END ! Nr of Hs =    0
  ATOM    H72    TYPE HA    CHARge    0.0    END ! Nr of Hs =    0
  BOND    C08    O81       BOND    C08    O82       BOND    C08    C07       BOND    C01    O11
  BOND    C01    C02       BOND    C01    O12       BOND    C02    C03       BOND    C02    H21
  BOND    C02    H22       BOND    C03    C04       BOND    C03    H31       BOND    C03    H32
  BOND    C04    C05       BOND    C04    H41       BOND    C04    H42       BOND    C05    C06
  BOND    C05    H52       BOND    C05    H51       BOND    C06    C07       BOND    C06    H62
  BOND    C06    H61       BOND    C07    H71       BOND    C07    H72

  { Note: edit these DIHEdrals if necessary }

    DIHEdral    O11    C01    C02    C03 ! angle 1
    DIHEdral    C01    C02    C03    C04 ! angle 2
    DIHEdral    C02    C03    C04    C05 ! angle 3
    DIHEdral    C03    C04    C05    C06 ! angle 4
    DIHEdral    C04    C05    C06    C07 ! angle 5
    DIHEdral    C05    C06    C07    C08 ! angle 6
    DIHEdral    C06    C07    C08    O81 ! angle 7
{
! DIHEdral    O81    C08    C07    H72 ! flexible dihedral ???      −93.68
! DIHEdral    O82    C08    C07    H72 ! flexible dihedral ???       86.30
  DIHEdral    O11    C01    C02    C03 ! flat ? (0 degrees = cis)        −9.19
! DIHEdral    O11    C01    C02    H21 ! flexible dihedral ???    −129.22
! DIHEdral    O11    C01    C02    H22 ! flexible dihedral ???      110.79
  DIHEdral    O12    C01    C02    C03 ! flat ? (180 degrees = trans)      170.85
! DIHEdral    O12    C01    C02    H21 ! flexible dihedral ???       50.82
! DIHEdral    O12    C01    C02    H22 ! flexible dihedral ???      −69.16
  DIHEdral    C01    C02    C03    C04 ! flat ? (180 degrees = trans)      180.12
! DIHEdral    C01    C02    C03    H31 ! flexible dihedral ???       60.03
! DIHEdral    C01    C02    C03    H32 ! flexible dihedral ???      −59.94
! DIHEdral    H21    C02    C03    C04 ! flexible dihedral ???      −59.88
  DIHEdral    H21    C02    C03    H31 ! flat ? (180 degrees = trans)      180.03
! DIHEdral    H21    C02    C03    H32 ! flexible dihedral ???       60.06
! DIHEdral    H22    C02    C03    C04 ! flexible dihedral ???       60.06
! DIHEdral    H22    C02    C03    H31 ! flexible dihedral ???      −60.03
  DIHEdral    H22    C02    C03    H32 ! flat ? (180 degrees = trans)      180.00
  DIHEdral    C02    C03    C04    C05 ! flat ? (180 degrees = trans)      180.42
! DIHEdral    C02    C03    C04    H41 ! flexible dihedral ???       60.36
! DIHEdral    C02    C03    C04    H42 ! flexible dihedral ???      −59.66
! DIHEdral    H31    C03    C04    C05 ! flexible dihedral ???      −59.51
  DIHEdral    H31    C03    C04    H41 ! flat ? (180 degrees = trans)      180.43
! DIHEdral    H31    C03    C04    H42 ! flexible dihedral ???       60.41
! DIHEdral    H32    C03    C04    C05 ! flexible dihedral ???       60.48
! DIHEdral    H32    C03    C04    H41 ! flexible dihedral ???      −59.58
```

```
  DIHEdral   H32   C03   C04   H42 ! flat ? (180 degrees = trans)     180.40
  DIHEdral   C03   C04   C05   C06 ! flat ? (180 degrees = trans)     180.14
! DIHEdral   C03   C04   C05   H52 ! flexible dihedral ???     −59.81
! DIHEdral   C03   C04   C05   H51 ! flexible dihedral ???      60.21
! DIHEdral   H41   C04   C05   C06 ! flexible dihedral ???     −59.75
! DIHEdral   H41   C04   C05   H52 ! flexible dihedral ???      60.30
  DIHEdral   H41   C04   C05   H51 ! flat ? (180 degrees = trans)     180.31
! DIHEdral   H42   C04   C05   C06 ! flexible dihedral ???      60.22
  DIHEdral   H42   C04   C05   H52 ! flat ? (180 degrees = trans)     180.27
! DIHEdral   H42   C04   C05   H51 ! flexible dihedral ???     −59.72
  DIHEdral   C04   C05   C06   C07 ! flat ? (180 degrees = trans)     180.35
! DIHEdral   C04   C05   C06   H62 ! flexible dihedral ???     −59.58
! DIHEdral   C04   C05   C06   H61 ! flexible dihedral ???      60.35
! DIHEdral   H52   C05   C06   C07 ! flexible dihedral ???      60.28
  DIHEdral   H52   C05   C06   H62 ! flat ? (180 degrees = trans)     180.36
! DIHEdral   H52   C05   C06   H61 ! flexible dihedral ???     −59.72
! DIHEdral   H51   C05   C06   C07 ! flexible dihedral ???     −59.74
! DIHEdral   H51   C05   C06   H62 ! flexible dihedral ???      60.33
  DIHEdral   H51   C05   C06   H61 ! flat ? (180 degrees = trans)     180.25
  DIHEdral   C05   C06   C07   C08 ! flat ? (180 degrees = trans)     179.68
! DIHEdral   C05   C06   C07   H71 ! flexible dihedral ???      59.67
! DIHEdral   C05   C06   C07   H72 ! flexible dihedral ???     −60.26
! DIHEdral   H62   C06   C07   C08 ! flexible dihedral ???      59.62
! DIHEdral   H62   C06   C07   H71 ! flexible dihedral ???     −60.39
  DIHEdral   H62   C06   C07   H72 ! flat ? (180 degrees = trans)     179.68
! DIHEdral   H61   C06   C07   C08 ! flexible dihedral ???     −60.32
  DIHEdral   H61   C06   C07   H71 ! flat ? (180 degrees = trans)     179.67
! DIHEdral   H61   C06   C07   H72 ! flexible dihedral ???      59.74
}

  { Note: edit these IMPRopers if necessary }

  IMPRoper   C08   C07   O81   O82 ! chirality or flatness improper      −0.01
  IMPRoper   C01   C02   O11   O12 ! chirality or flatness improper      −0.02

{
  IMPRoper   C02   C01   C03   H21 ! chirality or flatness improper      30.38
  IMPRoper   C03   C02   C04   H31 ! chirality or flatness improper      30.33
  IMPRoper   C04   C03   C05   H41 ! chirality or flatness improper      30.32
  IMPRoper   C05   C04   C06   H52 ! chirality or flatness improper     −30.33
  IMPRoper   C06   C05   C07   H62 ! chirality or flatness improper     −30.34
  IMPRoper   C07   C08   C06   H71 ! chirality or flatness improper     −30.37
}
{ Note: edit any DONOrs and ACCEptors if necessary }
```

```
{
  ACCEptor    O81    C08
  ACCEptor    O11    C01
  ACCEptor    O82    C08
  ACCEptor    O12    C01
}

END { RESIdue BS3 }
```
